# Strategies for Constructing College Students' Entrepreneurial Value Judgments Based on Educational Psychology

**DOI:** 10.3389/fpsyg.2021.657791

**Published:** 2021-06-23

**Authors:** Zhen Chen, Zimo Yang, Yuzhen Li, Yuanbing Liu, Xueling Jia, Jianhao Gao

**Affiliations:** ^1^School of Marxism, Northeastern University, Shenyang, China; ^2^Graduate School of Pan-Pacific International Studies, Kyung Hee University, Yongin-si, South Korea; ^3^The Graduate School of International Studies, Han Yang University, Seoul, South Korea; ^4^College of Teachers, Jiaxing University, Jiaxing, China; ^5^School of Education, Chongqing Normal University, Chongqing, China; ^6^School of Accounting Administration, Southwestern University of Finance and Economics, Chengdu, China

**Keywords:** educational psychology, entrepreneurial value, questionnaire survey, value judgments' construction strategies, college students

## Abstract

Against the background of economic globalization, the strategies for constructing college students' entrepreneurial value judgment are explored, providing college graduates with more employment options and thereby keeping up with the trend of the times. The documentary analysis and questionnaire survey methods are adopted to investigate contemporary college students' entrepreneurial value judgments, and the investigation results are organized. According to documentary materials, the discovered problems are analyzed to put forward strategies for constructing college students' entrepreneurial value judgments based on educational psychology. Results show that only 14.1% of college graduates choose to start a business; 48.7% do not understand or recognize the entrepreneurial values; 14.8% believe teaching activities on constructing entrepreneurial value judgments are insufficient, and the entrepreneurial atmosphere is lacking. Regarding the above investigation results, strategies for constructing college students' entrepreneurial value judgments are proposed, involving the construction environment, construction system, construction method, and construction mechanism. Hence, considering contemporary college students' entrepreneurial values, the proposed strategies for constructing college students' entrepreneurial judgments are suitable and valuable, providing a practical reference for enriching and perfecting the college innovation and entrepreneurship education systems.

## Introduction

Today, economic globalization has improved people's material lives significantly. While favorable conditions are created for talents' development, employment pressures come as the shadow follows the form. According to a survey, China's college graduates in 2019 are expected to reach 8.34 million, an increase of 140,000 compared with 2018, and a new record high (McArthur et al., 2017). As graduates demanding jobs increase continuously, job shortage has caused a severe supply-demand imbalance between the jobs and people waiting for employment, requiring society to create more jobs to meet the increasing job demand of college graduates (Ackerson and Stiles, 2018). Under such a background, entrepreneurship can enable college graduates to achieve employment; also, it can increase employment opportunities for society and drive more people to find jobs. Therefore, students' innovation and entrepreneurship have become the most important way to alleviate social employment pressure. However, continuously increasing the proportion of college students' self-employment and establishing correct entrepreneurial value judgments are problems requiring investigations and explorations.

College graduates are vital to China's economic development. Their employment is directly correlated to social development and college education. If college students cannot get jobs successfully after graduation, the investment return on education will be challenging to obtain. If they cannot find suitable jobs or employment methods, their general development requirements will not be met, which will harm the construction of a harmonious society (Grant and McDonald, 2018; Henseke, 2019). Correct value judgments can guide college students to participate in entrepreneurship teaching activities in the universities actively and consciously train students' entrepreneurial qualities and abilities. Also, correct value judgments can enrich the content of college students' entrepreneurship education and enhance college students' entrepreneurship education effectiveness. Practically, correct entrepreneurial value judgments can guide college students to embark on the path of entrepreneurship and guide the entrepreneurial process, and at the same time, ease the employment pressure on the society, making China develop toward a comprehensive well-off society (Wu and Song, 2019). Being internalized in people's spiritual levels, entrepreneurial value belongs to the theoretical category of value judgments and guides people's entrepreneurial behaviors and value selection. Educational psychology is closely connected to general psychology and pedagogy. Depending on the system of pedagogy, it researches how to cultivate the overall development of morality, intelligence, and physical education methods, promotes education and teaching, and achieves the optimal development of students with the best results (Gbadamosi, 2019; Sinthupundaja et al., 2020). As a psychology branch, educational psychology can develop many particular fields for studying educational issues, including instructional design, educational technology, curriculum development, organizational learning, special education, and classroom management (Williams, 2021). College students are in the stage of formation and development of entrepreneurial values. Guiding college students to establish correct values and educating college students' entrepreneurial value judgments are urgent problems in the entrepreneurial education process.

In summary, to provide more opportunities to college graduates to choose jobs and help them keep up with the trends of the times, helping college students construct innovative and entrepreneurial values according to the times and personal development needs has vital theoretical research values practical significance. The innovative points are: (1) using the sampling survey method to investigate and analyze the status quo of contemporary college students' entrepreneurial values; (2) understanding the measures, implementation, cultural atmosphere, and effects of innovation and entrepreneurship education in colleges and universities, to cultivate college students' entrepreneurial values, enrich the content of entrepreneurship education, strengthen the effectiveness of innovation and entrepreneurship education, and formulate targeted strategies to cultivate college students' entrepreneurial values. The research results are expected to provide a reference basis for formulating relevant measures for promoting college students' entrepreneurship education.

## Literature Review

Many scholars believe that entrepreneurs are an exceptional group. Moreover, college student entrepreneurs present unique personality traits, and these traits are a hot research topic. Gorgievski et al. (2018) studied the relationship between personal value judgments and entrepreneurial career intentions on a sample of 823 students from four European countries. They found that openness and self-improvement value judgments were positively correlated with entrepreneurial career intentions. Such a relationship was affected by entrepreneurial attitudes, self-efficacy, and social norms. Compared with Dutch, German, and Polish students, Spanish students showed lower entrepreneurial willingness and insufficient entrepreneurial enthusiasm. Lechner et al. (2018) investigated whether young people's job value judgments could predict their entrepreneurial ambitions and leadership ambitions, further explaining the impacts of gender differences on the value judgments that prevailed in people's daily works. Morales et al. (2019) assessed the cultural background of individual entrepreneurial values through the cultural dimension of egalitarianism to contextualize entrepreneurial behaviors. They found that personal value judgments were critical in explaining entrepreneurship, and egalitarianism reduced the impacts of self-improvement and openness on value judgment changes. Horst and Hitters (2020) understood the impacts of entrepreneurial strategy on the development of entrepreneurial identity and the construction of entrepreneurial knowledge. They found that entrepreneurial knowledge construction helped people understand the strategic works in the early stage of entrepreneurship. To analyze the connection between entrepreneurs' entrepreneurial mentality and entrepreneurial intentions, Alshebami et al. (2020) explored the components of entrepreneurial education and the Theory of Planned Behavior (TPB), namely, social norms, attitudes, and self-efficacy. They found that entrepreneurial education concepts had a significant impact on entrepreneurial attitudes.

Constructing college students' entrepreneurial value judgments is inseparable from the construction of entrepreneurial spirit. In the development process of China, entrepreneurial value construction is a vital link to strategic deployment. Tang et al. (2017) advanced the research on entrepreneurial value judgments by examining how the self-improvement and self-transcendence value judgments of Chief Executive Officers (CEOs) in Chinese state-owned and non-state-owned enterprises affected the relationship between Entrepreneurial Orientation (EO) and performance. The results found that CEOs with higher self-improvement in state-owned enterprises had a closer relationship with EO education performance, while CEOs with lower self-improvement in non-state-owned enterprises had a much closer relationship. He et al. (2019) reviewed the research on entrepreneurship caused by the unique system and culture behind the Chinese economy in recent years and found that entrepreneurship was one of the fundamental driving forces for sustainable economic development. Efforts should be made to encourage and promote entrepreneurial activities. Lang and Liu (2019) used an online open questionnaire to provide empirical evidence for entrepreneurial motivation theory research in a more detailed fashion entrepreneurial context. The results pointed out the developmental direction of fashion entrepreneurship courses based on the perspective of college students in fashion majors. Zhang et al. (2020) examined 668 tourism and hotel majors to explore whether entrepreneurship education would affect students' entrepreneurial intentions. The results found that support education positively impacted entrepreneurial intentions, and practice was the leading factor in forming entrepreneurial intentions, while theoretical improvement was an essential moderating factor. Cui et al. (2019) emphasized that Entrepreneurial Mindset (EM) was a new influencing factor of Entrepreneurship Education (EE). They conducted a sample survey regarding the weaknesses of current research on the association between EM and EE. Results found that entrepreneurship incentives had significantly enhanced students' entrepreneurial inspiration. They also emphasized the role of educational attributes, including the learning experience types, course types, and activity types.

The above analysis reveals that scholars worldwide mainly analyze entrepreneurial willingness and entrepreneurial spirit. Few analyses on entrepreneurial value from the perspective of psychology are reported. Therefore, regarding the trend of economic globalization, analyzing and researching the cultivation strategy of college students' entrepreneurial values based on educational psychology is vital for enriching and perfecting the innovation and entrepreneurship education system of college students under the background of “Mass Entrepreneurship and Innovation Initiative.”

## Methods

### Analytic Methods

**Figure 1:**
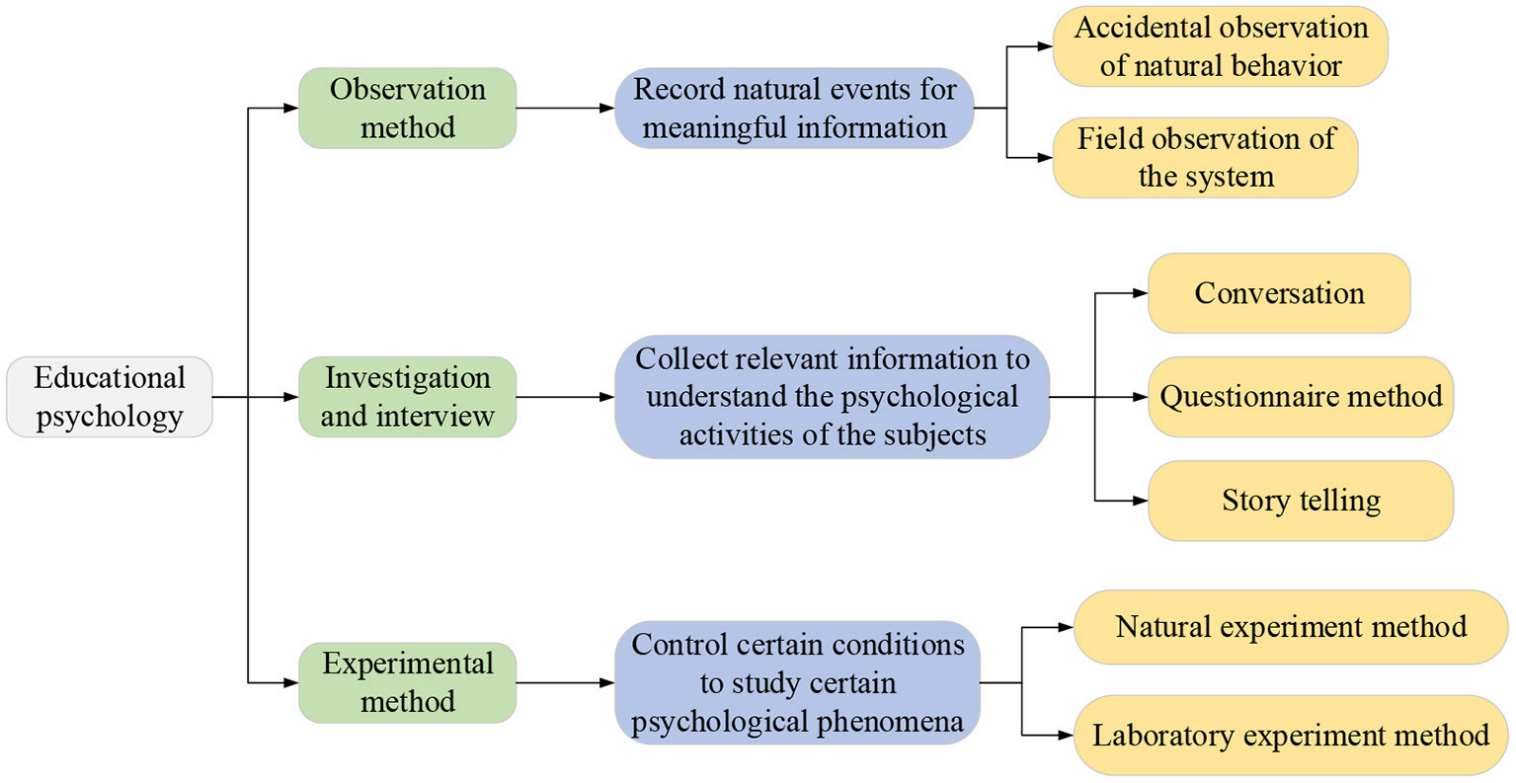
Research methods of educational psychology.

Here, the questionnaire survey method is adopted to analyze the impacts of educational psychology on college students' entrepreneurial value judgment construction and the strategies of constructing entrepreneurial value judgments. The research method of educational psychology is presented in Figure 1. According to the social cognition theory, the individual's value judgments, thinking styles, attitudes, and beliefs influence the individual's intentions and behaviors through the medium of cognition, including the guidance of individual positive attitudes and behaviors. The cognitive medium or program is affected by many factors of the individual, including personal temperament with substantial heritability and stability and the psychological factors with dynamic and plasticity (Biberhofer et al., 2019; Drobyazko et al., 2019). Such a psychological factor affecting the cognitive process and has plasticity can be seen as human capital or social capital. Through appropriate guidance, training, and reinforcement, individual value judgments can be developed, improved, and enhanced. Therefore, it is possible to develop and nurture entrepreneurial college students' psychology and ideas to change their cognition, promoting their thinking styles and value orientations. While students choose to start businesses independently, they can produce healthy, positive, and positive entrepreneurial attitudes and behavioral tendencies that are conducive to self-development and social development. Few works have explored the relationship between personality traits and social networking sites; instead, they focus on personality changes under the influence of social networking sites. Social media have changed the way people communicate with friends, as well as the way suppliers communicate with consumers (Li et al., 2019; Shen et al., 2019). Therefore, three common social platforms: Line, Facebook, and WeChat, are utilized for investigations. This is extremely important for guiding college students to establish the correct entrepreneurial values and promoting the sound development of the entrepreneurial ecosystem.

### Theoretical Foundation

#### Analysis of the Formation Characteristics of Contemporary College Students' Entrepreneurial Values

**Figure 2:**
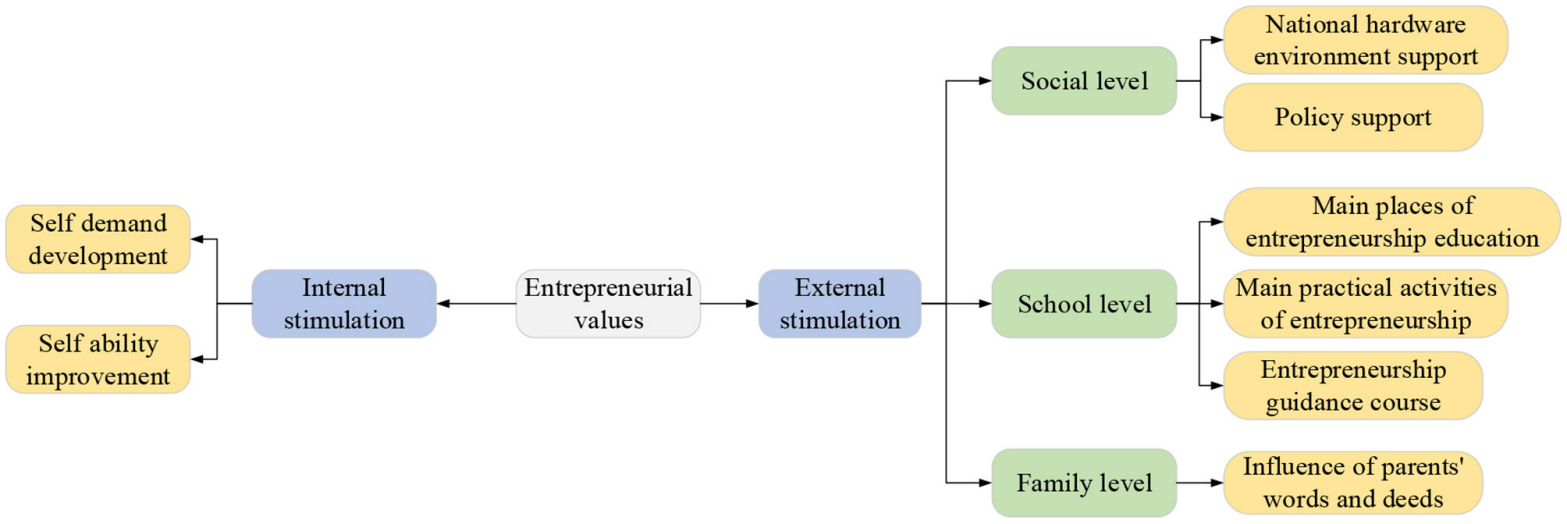
Analysis of the formation characteristics of contemporary college students' entrepreneurial values.

Psychologists believe that many external and internal factors stimulate individual behaviors. Essentially, behaviors are to satisfy individuals' internal or external needs. Motivation is the internal driving force of entrepreneurs to carry out entrepreneurial activities (Piva and Rossi-Lamastra, 2018; Link and Sarala, 2019). When college students start businesses, this motivation is affected by many factors, including internal stimulation and external stimulation, as shown in Figure 2.

In terms of internal stimulation, from college students' internal needs, entrepreneurial behaviors are college students' wishes and visions (Gu et al., 2018; Chen, 2019). College graduates hope to use their professional skills and knowledge to transform theories into reality. Under this trend, college graduates utilize their entrepreneurial ideas to carry out entrepreneurial practice activities, hoping to exercise their abilities from all aspects of thinking, emotion, and perseverance and then form entrepreneurial values.

External stimulation plays a vital role in repairing and developing college students' entrepreneurial values, which usually includes three levels: the society, the school, and the family (Baek, 2018; Syam et al., 2018; Qiao and Huang, 2019). At the social level, China has created an excellent hardware environment for college students to start their businesses, with policy supports in personnel agency fees, social security subsidies, and entrepreneurship training. Indeed, while obtaining venture capital supports, all supporting institutions will strictly inspect the entrepreneurs; if the entrepreneurs cannot meet the requirements, they cannot obtain corresponding investments. Therefore, social needs will inevitably promote college students to strengthen their personality cultivation and meet social development needs. The development and improvement of college students' entrepreneurial values are promoted through education courses at the school level. At the family level, family education is the key to forming college students' personalities, which also supplements entrepreneurial values. Only with their parents' support can college students' entrepreneurial behaviors be effectively carried out, making students more confident.

#### Analysis of the Influencing Factors of Educational Psychology on College Students' Entrepreneurial Values

Under the rapid economic development, more college graduates face job choices. College students have begun to invest in entrepreneurial practice activities, and the entrepreneurial values of college students have become one of the essential indicators to measure the quality of college students' entrepreneurial education (Lingappa et al., 2020). Educational psychology can effectively regulate and change college students' psychological cognition, affecting students' entrepreneurial attitude, intention, value judgment, and behavior. Educational psychology improves the relationship between entrepreneurial college students, the team and the entrepreneurs, and the external environment. A harmonious and progressive organizational culture and entrepreneurial ecological environment can be established, enhancing the moral responsibility and professional ethics of college entrepreneurs (Zhang et al., 2020). While college students' recognition and satisfaction with the entrepreneurial values are improved, their sense of identity and satisfaction with the organization, society, and the nation can be enhanced further.

Indeed, applying educational psychology to college entrepreneurs' educational management can change the practical problems encountered by college students in entrepreneurship and the relationship among college students, society, and the nation. When graduating college students encounter difficulties in entrepreneurial practice, they regularly adjust their behaviors to make their personal development consistent with social development needs to implement core socialist values (Apriana et al., 2019; Nuringsih et al., 2019). Therefore, while college entrepreneurs gain more competitive advantages in employment, they consciously practice social value judgments.

### Survey Design

A self-compiled questionnaire is utilized to investigate the current situation of college students' innovative entrepreneurial values empirically, in an effort to comprehensively understand college students' innovative entrepreneurial values and construct college students' innovative entrepreneurial values in a targeted manner. The questionnaire survey can understand the status quo, issues, and influencing factors of college students' innovative and entrepreneurial values, explore the cultivation strategies with shared and regional characteristics, and help college students develop innovative and entrepreneurial values. Three primary processes are involved in the questionnaire design. First, the relevant literature and data are reviewed. Previous works on strategies for college students' value judgment construction are reviewed. Data obtained from different channels are collected, sorted, and analyzed. While ensuring that the questionnaire is reasonable, the relevant variables involved in this questionnaire survey have also been initially determined. Second, the first draft of the questionnaire is designed. According to the literature review and data processing, the questionnaire is initially designed, and then the rationality of the indicator design is solicited from experts and scholars in the academic field. The first draft of the questionnaire is determined according to their revision opinions. Finally, the first draft of the questionnaire is revised.

The questions are proposed after relevant designs. A preliminary test is conducted on the questionnaire in a small range. In-depth corrections and adjustments are made according to the problems reflected in the preliminary test. After final revision, the questionnaire includes multiple dimensions such as career selections and factors, cognition and attitudes of entrepreneurial values, and constraints on entrepreneurial values (Danish et al., 2019). In this questionnaire, the entire questionnaire design, distribution, and data collection process does not involve privacy. The entire questionnaire survey is conducted with the consent of the participants (no <18 years old). The questionnaire is not open to the public and is only used for research purposes.

### Survey Authenticity Analysis

The survey authenticity is analyzed from three perspectives: the professionalism of the survey, the randomness of the data, and the scientific nature of the data.

First, the professionalism of the survey. Some third-party institutions or college student volunteers are invited to participate in this survey. These third-party participants must be screened and trained in advance, emphasizing the precautions and the significance of this survey and ensuring that they can be severe and responsible. Hence, the authenticity of the data is guaranteed.

Second, the randomness of the data. To enhance the rigor of this survey, three representative local undergraduate colleges and universities are selected, where equivalent questionnaires are issued for sample surveys. Each participant shall answer the questionnaire on-site in 15 min. Afterward, survey interviews will be conducted based on the questionnaire results, thereby ensuring the effectiveness of the questionnaire.

Third, the scientific nature of the data. After the survey is finished, the collected information will be sorted and analyzed to remove the useless information. Then, these data are integrated and summarized, which can intuitively reflect the current problems. Accordingly, some suggestions are put forward to provide a guarantee for formulating a scientific and reasonable basis. The reliability verification is performed to determine the validity of our data. Also, statistics, psychology, and other interdisciplinary approaches are employed to ensure the scientific nature of data analysis.

### Survey Data Statistics and Evaluation

The questionnaire's quality is inspected to verify the reliability and stability of the questionnaire data and indicator system related to college students' entrepreneurial values. The reliability coefficient used here is Cronbach's α (Dadfar and Lester, 2017), verifying the internal consistency between each question's scores in the questionnaire. Here, SPSS24.0 software evaluates the α reliability coefficient. Results indicate that the average value of Cronbach's α of the questionnaire is 0.843, indicating the questionnaire designed has strong internal consistency, stability, and validity, which is a reasonable and adequate questionnaire. Hence, this questionnaire can be used for the following research.

Students from three local universities are included to diminish this survey's deviation and enhance its scientificity and representativeness. Three popular social platforms, namely, Line, Facebook, and WeChat, are utilized for the sampling questionnaire survey. The total number of questionnaires is 843. A total of 798 questionnaires are returned, with a response rate of 94.66%, indicating that this questionnaire survey is an adequate investigation. The statistics of 798 valid questionnaires are carefully reviewed to ensure the validity of the questionnaire survey further. Questionnaires with obvious problems are eliminated. In the meantime, to minimize the error of the questionnaire, the number of questionnaires distributed in these three universities is kept the same. Two hundred and fifty-three valid questionnaires are taken from each college, totaling 759 valid questionnaires.

## Result

A questionnaire survey is performed on the entrepreneurial values of college students. The basic information and conditions are summarized in Table 1.

**Table 1:** Analysis of survey subjects' backgrounds.

		**The number of people counted**	**Percentage (%)**
Gender	Male	357	47.04
	Female	402	52.96
Grades	First year in college	318	41.90
	Second year in college	235	30.96
	Third year in college	129	17.00
	Fourth year in college	77	10.14
Types of majors	Economic management	182	23.98
	Science and engineering	258	33.99
	Humanities and social science	271	35.70
	Sports and arts	30	3.95
	Agriculture and medicine	18	2.37
Native place	Urban area	312	41.11
	Rural area	447	58.89

Table 1 suggests that females occupy most of this questionnaire survey, accounting for 52.96%; however, the ratio of males to females tends to be balanced overall. In terms of grade distribution, the survey subjects are mainly first grade, second grade, and third-grade students, with fewer fourth-grade students. This may be due to factors such as graduates are busy looking for jobs or preparing for post-graduate exams, studying abroad, and other factors that make them challenging to participate in the questionnaire survey. In terms of significant distribution, college students majoring in the humanities and social sciences, science and engineering, and economic management account for 35.70, 33.99, and 23.98%, respectively, consisting of the vast majority of the surveyed population. College students majoring in sports, art, agriculture, and medicine account for a tiny proportion, which may be related to the nature and significant settings of the universities surveyed. In terms of native place distribution, the number of surveyed students from rural areas accounts for 58.89%, while the urban population accounts for 41.11%, which may be associated with the local economic development.

### Career Choice and Factor Analysis

**Figure 3:**
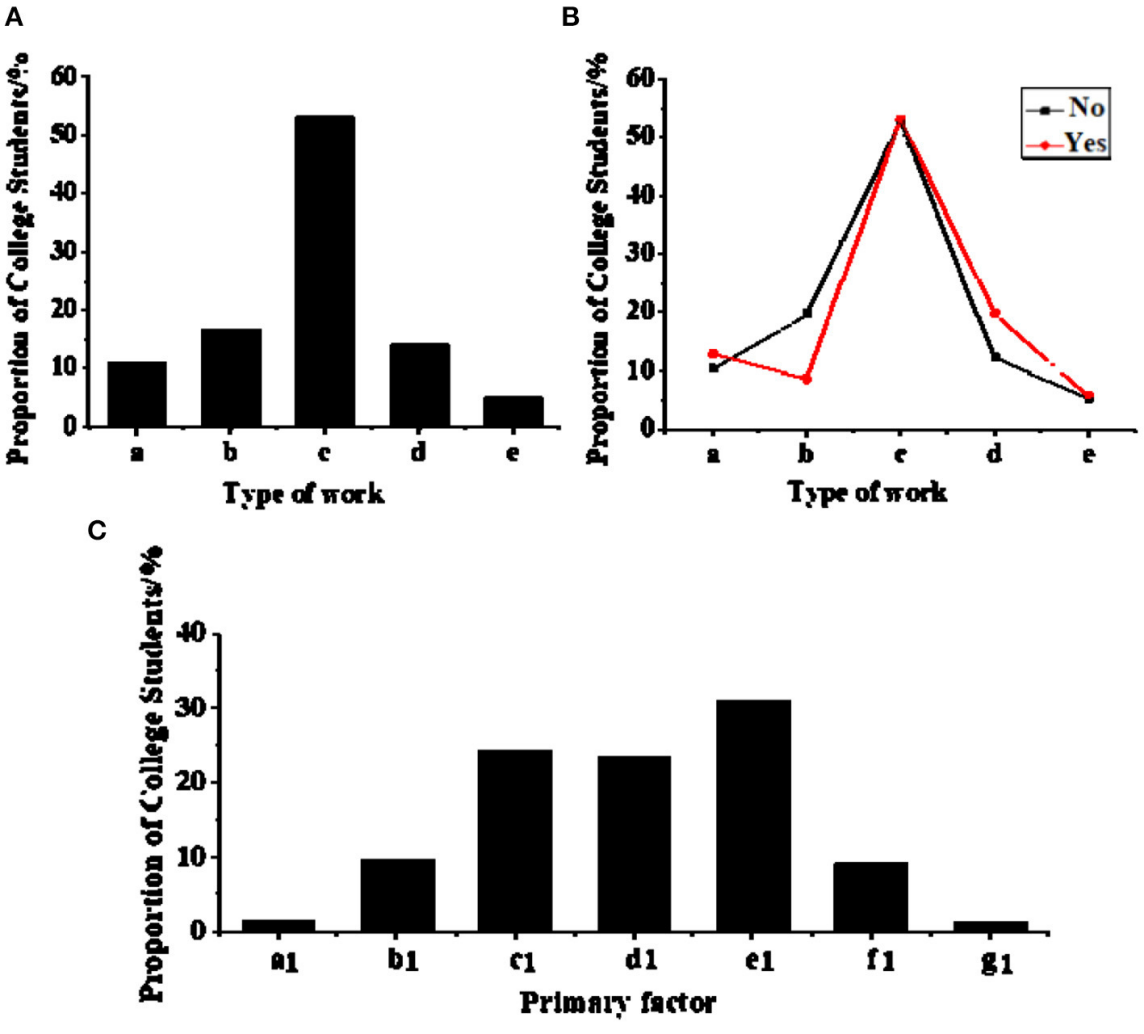
Career choice and factors' statistical analysis [**(A)**. College students' career choices. a. Party and government organs; b. Public institution; c. Company; d. Self-employed; e. Else **(B)**. Whether students have received innovation and entrepreneurship education when choosing a career. **(C)**. The primary factors in choosing a career. a1. Social needs; b1. Personal value realization; c1. Development space; d1. Personal interests; e1. Pay level; f1. Professional counterparts; g1. Else].

Statistics on college students' career choices are shown in Figure 3A. More than 50% of college students hope to work in a company or enterprise, which accounts for the most significant proportion. Other career choices are education and scientific research institutions, party and government organs, independent entrepreneurship, and other jobs. Further analysis reveals that 85.9% of college students choose “employed full-time jobs,” while college students who start businesses account for only 14.1%. From the perspective of whether students have received innovation and entrepreneurship education in choosing careers, as shown in Figure 3B, the main differences are “education and scientific research institutions” and “independent entrepreneurship.” Without innovation and entrepreneurship education, the proportions of college students who choose “education and scientific research institutions” and “independent entrepreneurship” are 19.6 and 12.2%, respectively. After receiving innovation and entrepreneurship education, students' proportions choosing “education and scientific research institutions” and “independent entrepreneurship” are 8.6 and 19.8%, respectively. This result shows that innovation and entrepreneurship education significantly impact college students' choice of independent entrepreneurship. The primary factors in choosing a career are analyzed. The top three are the pay level, development space, and personal hobbies (Figure 3C). Other factors account for less.

### Investigation and Analysis of Cognition and Attitude on Entrepreneurial Values

**Figure 4:**
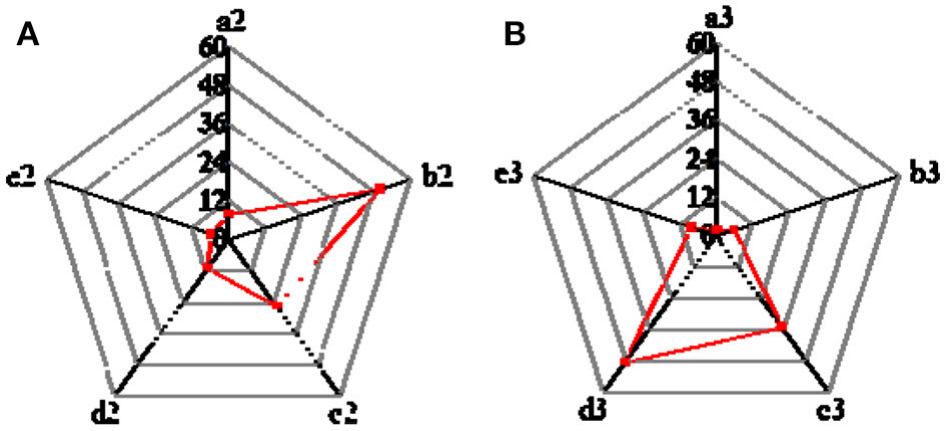
College students' cognition and attitudes on entrepreneurial values [**(A)**. College students' recognition of entrepreneurship. a2 Science and technology projects; b2. Entrepreneurial activities based on Innovation; c2. Open work; d2. Enterprise and company; e2. Else **(B)**. College students' mastery of entrepreneurship-related policies and measurements. a3. Very well; b3. Understand; c3. General; d3. Do not understand; e3. Don't care].

The survey and analysis results of the cognition and attitude of college students on entrepreneurial values are shown in Figure 4. In terms of entrepreneurship's cognition, 50.2% of college students regard the meaning of “innovation and entrepreneurship” as “entrepreneurial activities based on innovation” (in a broad sense), 10.9% consider “innovation and entrepreneurship” as “founding a business or company based on innovation” (in a narrow sense). Therefore, from an overall perspective, the number of college students in Guangxi Province who have a clearer understanding of the meaning of “innovation and entrepreneurship” is relatively large, accounting for 61.1%; college students who regard “innovation and entrepreneurship” as other meanings account for 38.9% (Figure 4A).

Five options are set up: “very well,” “understand,” “general,” “do not understand,” and “do not care,” to clarify the degree of college students' understanding of policies and measurements related to innovation and entrepreneurship. As shown in Figure 4B, 35.3% of college students choose “general,” and 48.7% choose “do not understand.” Thus, most college students have perfunctory attitudes toward entrepreneurial knowledge.

### Analysis of Entrepreneurial Value's Restricting Factors

**Figure 5:**
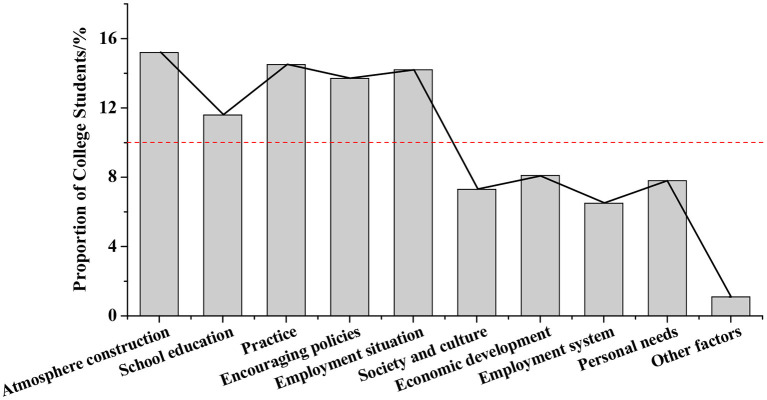
Analysis of entrepreneurial value's restricting factors.

Factors restricting the development of college students' innovative entrepreneurial values are shown in Figure 5. Five restricting factors account for more than 10%, which are atmosphere creation (15.2%), practice (14.5%), employment situation (14.2%), encouragement policies (13.7%), and school education (11.6%). Other restricting factors account for <10%, including social culture (7.3%), economic development (8.1%), employment system (6.5%), personal needs (7.8%), and other factors (1.1%).

**Figure 6:**
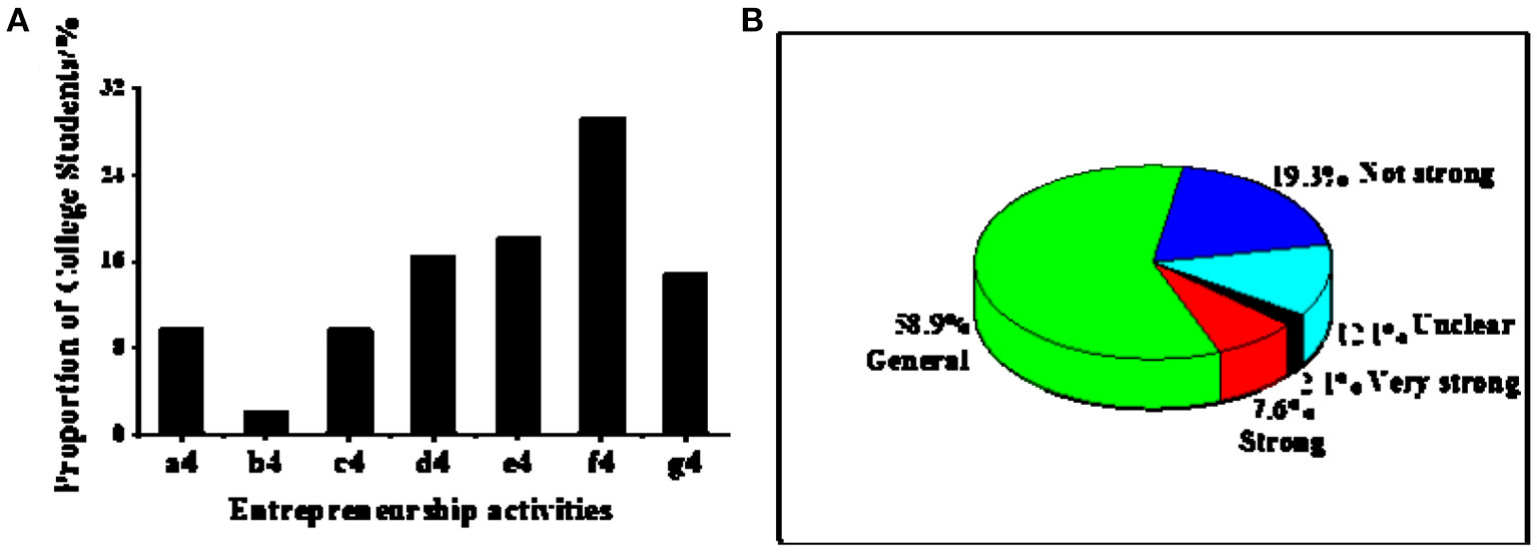
Analysis of the development of entrepreneurial activities in universities and atmosphere analysis [**(A)**. Analysis of the current situation of entrepreneurial activities in universities. a4. Unclear; b4. Else; c4. Related practice; d4. Entrepren-eurial base; e4. Related speeches; f4. Related lectures; g4. Related courses; **(B)**. Investigation on the degree of the entrepreneurial atmosphere in universities].

The current situation of innovation and entrepreneurship-related activities in surveyed universities is shown in Figure 6A. About 29.3% of college students reflect that the primary form of innovation and entrepreneurship activities in schools is lectures. Other activities are speeches (18.2%), entrepreneurial bases (16.5%), and courses (14.8%). Also, 1.9% of college students report other forms, and 9.7% do not know this. In general, 14.8% of college students believe that the primary form of innovation and entrepreneurship activities in their schools is teaching activities, while 85.2% choose non-teaching activities. A further survey of the entrepreneurial atmosphere of college students is shown in Figure 6B. The “general atmosphere” accounts for the largest proportion (58.9%), followed by the “unclear atmosphere,” accounting for 19.3%. Both the “strong atmosphere” and the “very strong atmosphere” account for <10%.

## Discussion

### Analysis and Discussion on the Status Quo of College Students' Values

College students' entrepreneurial values are investigated. Overall, a small proportion of college graduates choose to start a business; almost a half does not understand or recognize the entrepreneurial values; college graduates believe teaching activities on constructing entrepreneurial value judgments are insufficient, and the entrepreneurial atmosphere is lacking. There are two kinds of innovation and entrepreneurship activities in colleges and universities: teaching activities and non-teaching activities (Gorgievski et al., 2018; Komarova et al., 2019). A minority of college students believe that the main form of innovation and entrepreneurship activities in their schools is teaching activities; in contrast, most college students believe that the main form of innovation and entrepreneurship activities in their schools is non-teaching, that is, extracurricular activities. This reveals that colleges and universities focus on educating college students' innovation and entrepreneurship values through non-teaching activities, such as lectures, training, competitions, and on- and off-campus practices. This result coincides precisely with the findings of Gbadamosi (2019) and Sinthupundaja et al. (2020). The innovation and entrepreneurship education activities in colleges and universities have achieved some fruitful results. However, to reinforce college students' values, innovation and entrepreneurship education must be strengthened continuously; this is consistent with the findings of Deng et al. (2021).

### Path and Strategies for Constructing College Students' Entrepreneurial Value Judgments Based on Educational Psychology

**Figure 7:**
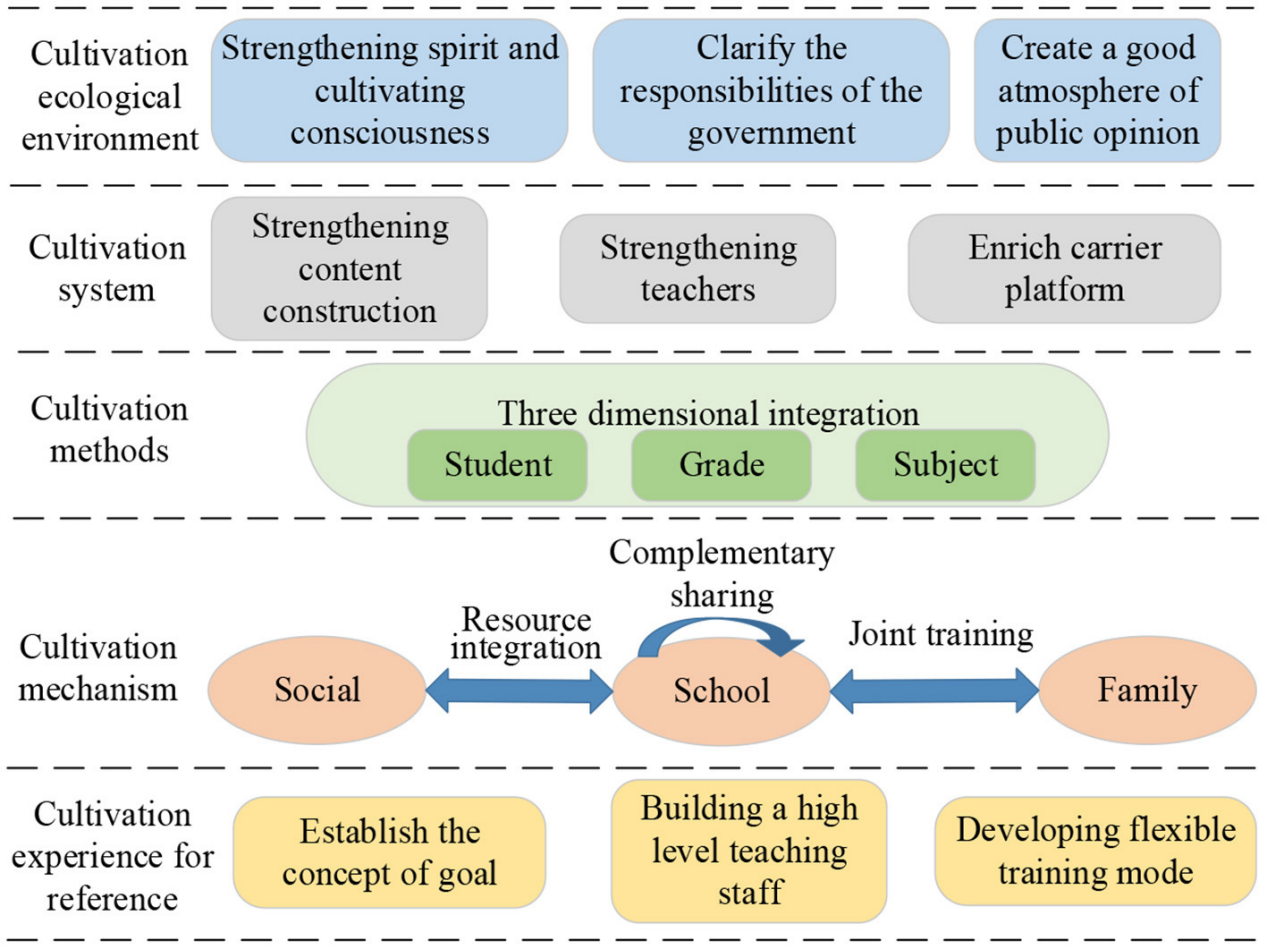
Framework of strategies for constructing college students' entrepreneurial value judgments.

The above investigation results suggest strategies for constructing college students' entrepreneurial value judgments, a systematic project involving various aspects, including the construction environment, construction system, construction method, and construction mechanism. Simultaneously, coordination and cooperation among the society, family, and universities are also needed (Biberhofer et al., 2019). Strategies for constructing college students' entrepreneurial value judgments are shown in Figure 7.

Strategies for college students' entrepreneurial value judgment construction are researched from multiple angles:

First, the construction and promotion of college students' entrepreneurial values require an excellent ecological environment. Entrepreneurship education in Chinese universities has started late, and the construction awareness, government responsibilities, and public opinion atmosphere have inhibited the development of college students' entrepreneurial spirit to some extent (Herdjiono et al., 2017). Therefore, college students' entrepreneurial value judgments should be constructed by strengthening the awareness of value judgment, clarifying the government's responsibilities, and creating a good public opinion atmosphere (Chen, 2019).

Second, the entrepreneurial values of college students should be cultivated comprehensively. Entrepreneurs can enhance their role identification, maintain a positive entrepreneurial attitude, stimulate their creativity, make innovations, and enhance their energy efficiency awareness (Feng and Chen, 2020). To cultivate college students' entrepreneurial values, it is necessary to build a comprehensive training system for colleges and universities, including content construction, teacher construction, and platform carrier construction. In terms of content construction, college students' senses of responsibility, adventure and exploration, active learning, and perseverance should be strengthened. In terms of teacher construction, a team of professional scholars who specialize in ideological and political theory courses and theoretical research on innovation and entrepreneurship should be constructed; meanwhile, teachers, professional counselors, and part-time personnel should also engage in the construction of innovation and entrepreneurship, making them cooperate scientifically, clarifying their duty divisions, thereby playing and establishing a faculty team that demonstrates the role of educators. Finally, platform carriers, such as ideological and political education classrooms, university science parks, and “Internet+” college students' innovation and entrepreneurship competition, should be established and improved to provide college students with a complete entrepreneurial value construction platform.

Third, according to different stages, levels, and needs of college students, targeted development should be implemented, focusing on the combination of theory and practice. A “three-dimensional integration” approach should be created to fostering an entrepreneurial value based on the dimensions of grade, student, and subject. In the grade dimension, the first year is a transitional stage; the first-grade students have strong plasticity and variability. Basic theoretical courses can be offered to teach students the necessary theoretical knowledge. The second-grade students have adapted to the study and life in the universities; thus, their entrepreneurial thinking and ability can be educated through selective participation in club activities and social activities. The world outlook, life outlook, and value judgments of third-grade and fourth-grade students have formed. Through targeted innovation and entrepreneurship courses, the continuous construction of entrepreneurial value for college students will lay the foundation for college students' future entrepreneurship and employment. In the student dimension, the individual differences of students should be values. Each student's ideas should be respected, allowing students to communicate and learn in an open environment to feel the joy of learning and ultimately maximize their subjectivity and enthusiasm. In the subject dimension, by integrating innovative entrepreneurial values into the original professional education of college students, students can apply professional theoretical knowledge to practice; hence, their practical ability and quality can be significantly improved.

Fourth, a diversified and efficient linkage construction mechanism is established by analyzing the relationship among society, family, colleges and institutions, systems and institutions, and various factors and links, which aims to promote the orderly operation of college students' innovative and entrepreneurial activities and mobilizing college students' enthusiasm for innovation and entrepreneurship. Finally, college students' innovative entrepreneurial value judgments can be constructed. Between colleges and universities, by combining their realities, forming a relationship of assistance, and building a sharing mechanism, the sharing of resources cultivated by an innovative entrepreneurial value between colleges and universities can be further improved. The linkage between universities and society should be promoted. The new media can maximize the benefits of new media technology and further develop college students' innovative entrepreneurial values. In terms of colleges and families, families play a guiding role in college students' innovation and entrepreneurship, an essential resource for constructing innovative entrepreneurial values in colleges and universities. A joint construction mechanism between colleges and families can be constructed by strengthening the connection between colleges and families of college students. Therefore, colleges and universities can establish a stable relationship with students' families, thereby constructing college students' innovative entrepreneurial value judgments more targeted.

Fifth, lessons can be drawn from other countries' experiences in constructing college students' entrepreneurial value judgments. The value judgment's construction strategies of Chinese college students' entrepreneurial values can be improved by establishing the concept of strategic goals, building a high-level faculty, and developing a flexible training and education model.

## Conclusion

Under the trend of “Mass Entrepreneurship and Innovation Initiative,” the cultivation strategies of college students' entrepreneurial values are researched based on educational psychology. Moreover, the status quo of college students' entrepreneurial values is investigated. Results reveal that innovation and entrepreneurship education in colleges and universities is effective; meanwhile, it is also necessary to continuously strengthen innovation and entrepreneurship education, especially college students' innovative and entrepreneurial values. Therefore, according to the current situation, complementary strategies for cultivating college students' entrepreneurial values have been formulated, providing a reference for the later research work. Indeed, there are some deficiencies in the research work. First, the survey subjects are students sampled from three local undergraduate colleges, which may cause regional differences in the results, resulting in substantial limitations. Therefore, in the future, the sample number and the number of regions can be increased to improve the results' credibility and provide a basis for students' mental health assessment in higher vocational colleges.

## Data Availability Statement

The raw data supporting the conclusions of this article will be made available by the authors, without undue reservation.

## Ethics Statement

The studies involving human participants were reviewed and approved by Northeastern University and Jiaxing University Ethics Committee. The patients/participants provided their written informed consent to participate in this study. Written informed consent was obtained from the individual(s) for the publication of any potentially identifiable images or data included in this article.

## Author Contributions

All authors listed have made a substantial, direct and intellectual contribution to the work, and approved it for publication.

## Conflict of Interest

The authors declare that the research was conducted in the absence of any commercial or financial relationships that could be construed as a potential conflict of interest.
